# Geiger-Mode Avalanche Photodiode Arrays Integrated to All-Digital CMOS Circuits

**DOI:** 10.3390/s16040495

**Published:** 2016-04-08

**Authors:** Brian Aull

**Affiliations:** Massachusetts Institute of Technology Lincoln Laboratory, 244 Wood St, Lexington, MA 02420, USA; aull@ll.mit.edu; Tel.: +1-781-981-4676

**Keywords:** imagers, imaging, avalanche photodiodes, Geiger-mode avalanche photodiodes, lidar, ladar, wavefront sensing, photon counting

## Abstract

This article reviews MIT Lincoln Laboratory's work over the past 20 years to develop photon-sensitive image sensors based on arrays of silicon Geiger-mode avalanche photodiodes. Integration of these detectors to all-digital CMOS readout circuits enable exquisitely sensitive solid-state imagers for lidar, wavefront sensing, and passive imaging.

## 1. Introduction

The MIT Lincoln Laboratory (Lexington, MA, USA) has been a world leader in the development of specialized high-performance charge-coupled-device imagers (CCDs) [1]. CCDs have outstanding performance and support advanced functions such as charge-domain image stabilization, electronic shuttering, and blooming control. The ultimate sensitivity limitation of a CCD is set by the readout noise of the output amplifier that senses the charge packets and converts them to analog voltage levels. The faster the readout rate, the more severe the readout noise penalty. This sensitivity limitation becomes important in photon-starved applications, such as night vision or high-temporal-resolution imaging.

Interest in such scenarios lead to Lincoln’s development of photon-counting image sensors, primarily based on arrays of custom-fabricated Geiger-mode avalanche photodiodes (GMAPDs) integrated with all-digital CMOS readout circuits. The term “photon counting” is used broadly here to mean that each photon arrival is digitally recorded by the pixel circuit. The pixel could be designed to either time stamp photons or count them. While the primary focus of this article is silicon GMAPDs [2], Lincoln Laboratory has developed GMAPD arrays based on compound semiconductors sensitive at wavelengths ranging from 1.06 μm [3] to the mid-wave infrared [4].

The principal advantage of a photon counting image sensor is that it eliminates readout noise. Because digitization occurs within the pixel, there is no analog circuitry in the readout path, and therefore no analog circuit noise. This means that there is no readout noise penalty for operating at high frame rates, using short integration times, or dividing the incoming light into multiple spectral or spatial channels. In-pixel digitization therefore enables high-frame-rate imaging [5,6], multispectral imaging [7], and spatial oversampling [8].

A second advantage of a photon-counting image sensor is that it facilitates in-pixel time-to-digital conversion. The ability to digitally time stamp individual photon arrivals is an enabler for exquisitely sensitive lidar imaging systems. The GMAPD arrays can also be used as laser communication receivers in systems with challenging link budgets. Use of pulse-position-modulated formats allows for multiple bits of information to be encoded in a single transmitted photon [8].

The integration of GMAPDs with digital CMOS can take advantage of Moore’s Law scaling and 3D integration to incorporate on-focal-plane processing functions such as imaging stabilization, spatial filtering, change detection, and tracking of objects of interest without mechanical scanning.

Potential disadvantages of GMAPDs include afterpulsing and crosstalk. Afterpulsing denotes false detection events triggered by carriers generated in previous events. To avoid afterpulsing the avalanche photodiode (APD) must be debiased for a sufficient time after an event to give such residual carriers time to recombine or be collected. Since the APD is unresponsive during this time, this leads to blocking loss and causes the count rate to saturate at high optical fluxes. Crosstalk is mediated by hot-carrier light emission by the avalanche process and is discussed at the end of Section 4.

## 2. Geiger-mode APDs and Photon-to-Digital Conversion

There are many types of detectors that have single-photon sensitivity, including photomultiplier tubes, image intensifiers, superconducting nanowires, and CCDs with avalanche gain registers [9]. GMAPDs, also known as single-photon avalanche detectors or SPADs [10], have a combination of advantages over these other photon-counting technologies. It is an all-solid-state device technology, scalable to many pixels, capable of room-temperature or thermoelectrically-cooled operation, and as noted already, amenable to on-focal-plane digitization and processing.

An avalanche photodiode is a *p*-*n* junction diode whose doping profile supports high electric fields near the junction at operational bias. A photoelectron or photohole created by the absorption of a photon is accelerated to sufficient energy to initiate a chain of impact ionization events, creating offspring electron-hole pairs and leading to internal gain. Once this avalanche process is underway, there is a competition between carrier generation and carrier extraction. Below the avalanche breakdown voltage, carriers are being extracted faster than they are being generated. The current flow decays, leading to self-termination of the avalanche process. This gives finite gain and therefore produces a photocurrent that is proportional to the intensity of the incident light. This is the traditional mode of operation used in optical communication receivers, known as linear mode. The gain is an increasing function of bias, and there is a random variation of gain that degrades signal-to-noise ratio.

The APD can also be operated above the avalanche breakdown voltage. When an avalanche starts, carrier generation predominates over extraction leading to exponential growth of the current. This growth is arrested by series resistance (in most cases mediated by space charge buildup). This mode of operation is known as Geiger mode, in which a single photon can initiate an avalanche that is self-sustaining. Of course, the APD must then be reset by reducing the bias to below breakdown long enough to terminate the avalanche, a process known as quenching.

**Figure 1:**
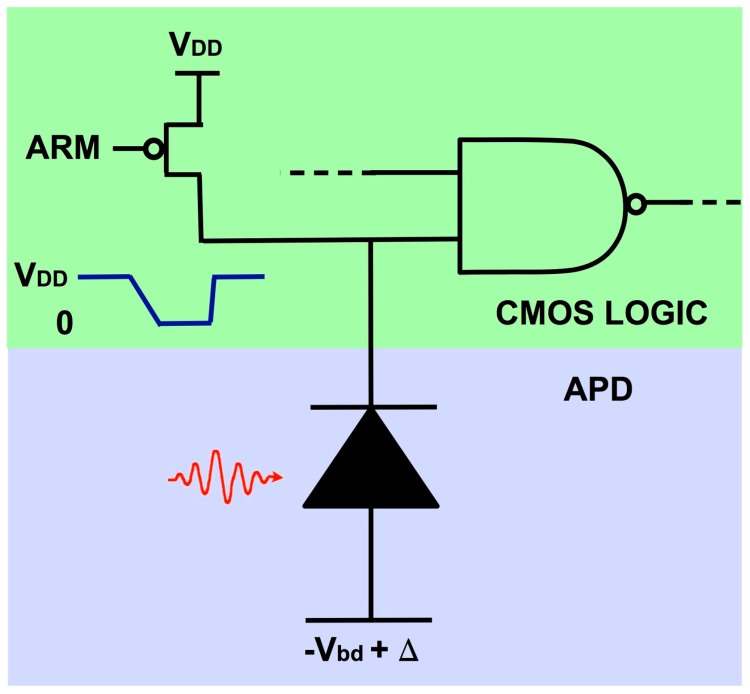
Photon-to-digital conversion. The GMAPD is biased so that it produces a CMOS-logic-compatible voltage pulse, enabling a direct connection to pixel logic.

The physics of Geiger-mode operation lends itself to a startlingly simple way of interfacing the GMAPD to a digital CMOS circuit: a direct connection to the input of a logic element, illustrated in Figure 1. The *p*-side of the APD is tied to a negative bias voltage slightly less in magnitude than the breakdown. The *n*-side is connected to a logic circuit that performs simple voltage sensing. Initially, the n-side of the APD is set to a logic-high voltage by briefly turning on a pull-up transistor. Once armed in this fashion, the APD has a reverse bias several volts above breakdown. When an avalanche is initiated, the APD turns on and then discharges its own parasitic capacitance. Once at or below breakdown, the avalanche terminates, leaving the *n*-side at a voltage close to a logic 0. The APD is effectively a CMOS-compatible digital element. Also, like a CMOS logic element, the APD draws current only when it is switching. This “photon-to-digital conversion” scheme has been the basis of all of Lincoln’s GMAPD imaging devices, with minor modifications. One can, for example, add a pull-down transistor to speed up the APD discharge, size the transistors in the sensing gate to adjust the triggering threshold, or cascode transistors to augment the voltage swing. Voltage sensing gives more timing latency and timing jitter than current sensing circuits [11]. This drawback has been more than offset by the advantages of the photon-to-digital conversion scheme: simplicity, robustness, and freedom from static power dissipation.

## 3. Fabrication and Integration with CMOS

**Figure 2:**
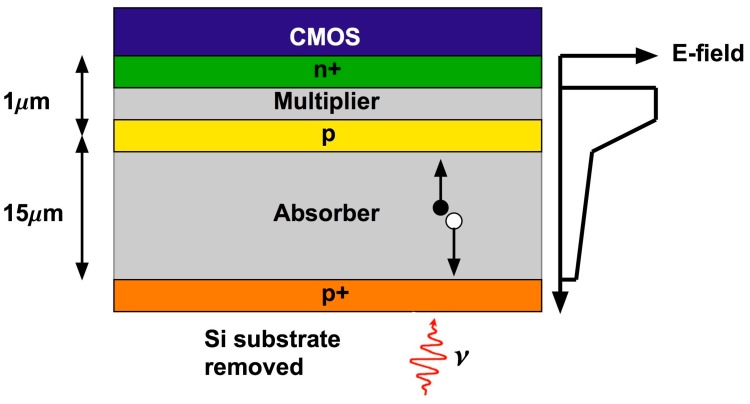
Simplified cross section showing pixel structure, field profile, and operation.

Lincoln Laboratory’s silicon APD arrays are fabricated in house in its Microelectronics Laboratory, currently on 200-mm silicon substrates. Typically, the substrate is heavily p doped, with a lightly p-doped epitaxial layer on which the APD structure is fabricated. The doping profile of the APD is defined by a series of patterned ion implantation steps. After back-end processing to make contact pads, the APD array is integrated with a CMOS readout circuit, using either bump bonding or a 3D-integration technique. During this process the opaque silicon substrate must be removed to enable back-side illumination. The back side is passivated with a p-doped contact layer. This layer is typically implanted and then activated using laser annealing, although molecular beam epitaxy has also been used to grow the contact layer. In many cases an antireflection coating and a sparse contact metal pattern is also added. A simplified cross section of the pixel structure is shown in Figure 2. The APD is a separate-absorber-multiplier structure, and the figure also shows the electric field profile at operational bias. A photon incident from the back side creates an electron-hole pair in the absorber. The hole is collected at the back side, and the electron drifts to the multiplier region, where the avalanche is initiated.

A number of investigators have demonstrated front-illuminated silicon GMAPD arrays using a monolithic device structure in which the photodiode is incorporated into the CMOS readout circuit using a standard CMOS foundry process [10]. This approach facilitates rapid and low-cost prototyping. Because of the thin device structure, monolithic CMOS APDs can time stamp photon detections with very low (tens of ps) timing jitter. However, they share pixel area with the readout circuitry and this limits fill factor. Moreover, fabrication using a standard CMOS process flow precludes customization of photodiode doping profiles and layer thicknesses. Photon detection efficiency is typically poor in the red and near-IR spectral regions. This is the reason why Lincoln has pursued back-illuminated APD arrays hybridized to foundry-fabricated CMOS.

Hybridization of compound semiconductor detector arrays (InGaAs or HgCdTe) is common. These detectors, however, are fabricated in layers heteroepitaxially grown on transparent substrates that serve as a mechanical support during bump bonding and device operation. Silicon APDs are fabricated on homoepitaxially grown layers on a substrate that is opaque at wavelengths of operation. The substrate must be entirely removed either before or after bump bonding, entailing difficulties associated with handling of a thin (15 μm) detector layer or with transfer of this layer to a transparent substrate.

The first GMAPD arrays made at Lincoln were hybridized by a technique known as bridge bonding. Individual CMOS die were epoxied to an APD wafer, providing immediate mechanical support, but with no electrical connection. After removal of the APD wafer substrate, large vias were etched through the detector layer between pixels to expose connection pads, and a metal strap patterned to complete the electrical connection. This technique worked but its use is limited to devices with coarse (>50 μm) pixel pitch and low fill factor [2].

**Figure 3:**
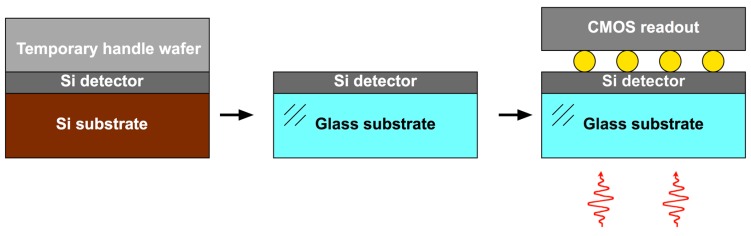
Transfer and bump bonding.

Another technique pursued is transfer and bump bonding [12], shown in Figure 3. Once the APD arrays are fabricated, the wafer is bonded to a temporary silicon handle wafer. The APD substrate is removed and then the device bonded to a transparent fused silica substrate. The temporary handle is removed, and then bump bonding to CMOS readout chips can be carried out in the same manner as for the heteroepitaxial detector materials. Both the temporary handle and the transparent substrate are bonded using oxide-oxide bonding with no adhesive. The transfer process provides APD arrays that can then be quickly bumped to any CMOS readout chip that matches the format and pixel pitch. It is, however, a relatively complex process because of the two-step transfer involved.

**Figure 4:**
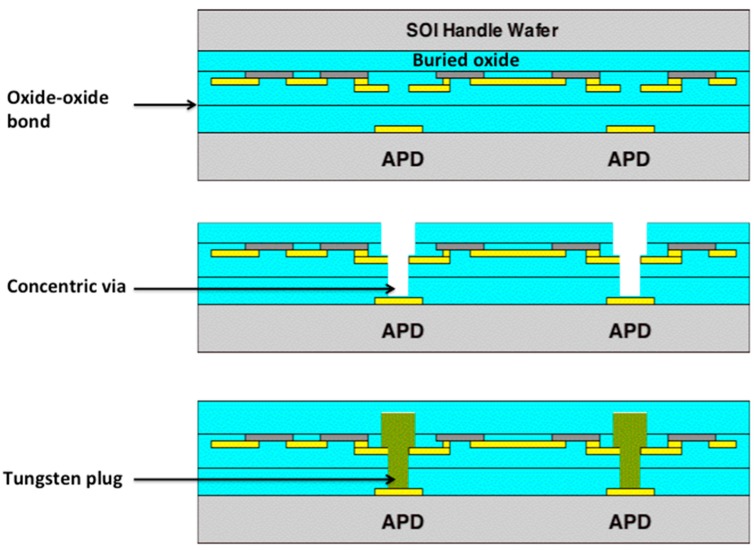
Steps in the Lincoln 3D-integration process to integrate the APD wafer (tier 1) with the first SOI CMOS wafer (tier 2).

**Figure 5:**
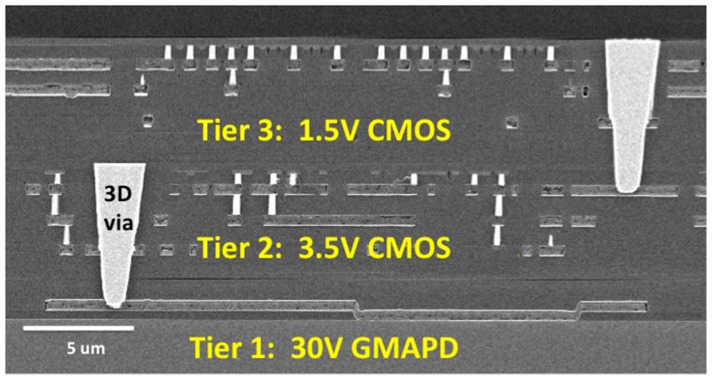
Cross-sectional SEM through a pixel of the 64×64 GMAPD array.

Lincoln Laboratory pioneered a 3D-integration process using its in-house fully depleted silicon-on-insulator (SOI) CMOS process. One of the first imager demonstrations was a 64 × 64-pixel GMAPD array for lidar [13]. The APD was integrated with two tiers of SOI CMOS readout circuitry. Figure 4 illustrates the process. After APD and SOI CMOS fabrication is complete, the APD wafer (tier 1) and the first CMOS wafer (tier 2) are precision aligned and oxide bonded face to face. Then the SOI handle wafer is removed, using the SOI buried oxide as an etch stop. Concentric vias are then etched through the oxide layers and filled with tungsten to interconnect the last metal of the SOI wafer with the top metal of the APD wafer. These tungsten plugs are micron-scale in diameter and only a few microns in height, much smaller than bump bonds or through-silicon vias used in some wafer stacking processes. The process can be repeated, adding the tier-3 CMOS wafer to the stack. Because the SOI handle wafer is removed, the process is amenable to multiple-tier structures. Figure 5 shows a cross-sectional micrograph of a pixel of the 64 × 64-pixel GMAPD lidar imager.

3D integration is an important technology direction for advanced image sensors. In the device structure shown in Figure 5, tier 1 contains the photodetectors, tier 2 has first-level signal conditioning circuits, and tier 3 has high-speed logic for photon time stamping. The ability to divide the pixel circuitry among multiple tiers enables a sophisticated pixel circuit to fit into a smaller area than would be possible for a conventional 2D-integrated circuit. A tier could be dedicated to image processing functions to extract information at the pixel level and reduce readout bandwidth. Having multiple tiers also enables the designer to combine multiple circuit or device technologies into a single integrated device, as in Figure 5.

**Figure 6:**
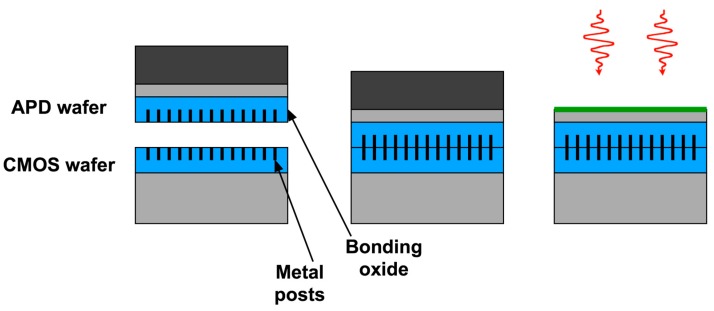
*Via-first* 3D integration.

The Lincoln 3D process is an example of a *via-last* process. The connection via from each tier of SOI CMOS down to the previous tier is made after wafer bonding and SOI handle removal. This approach is amenable to multi-tier structures, but requires dedicated full-wafer SOI CMOS for each tier, with the exception of tier 1. One can also use a *via-first* approach [14], illustrated in Figure 6. Metal posts (which perform the same function as bump bonds, but are much smaller) are patterned on each wafer and planarized along with the bonding oxide. When the wafers are bonded and then heated to strengthen the oxide-oxide bond, the posts expand and fuse. In a single step, therefore, mechanical bonding and electrical connection are achieved simultaneously. The CMOS wafer also functions as a permanent mechanical support for APD substrate removal, backside processing, and subsequent imager operation. The *via-first* approach allows for the use of any CMOS process, although it is not easily extendable to multi-tier structures.

## 4. Application to Lidar Imaging

The first application of Lincoln’s GMAPD arrays was optical flash radar for three-dimensional imaging. This is known as ladar or lidar, and we will use the second term. In a flash lidar system the scene is illuminated by a short laser pulse, and imaged onto an array of detectors, each of which measures photon arrival time, and therefore depth to the corresponding point in the scene. By using an array of detectors, one can avoid the mechanical scanning needed in single-detector systems. Because the available light is divided among many pixels, however, the average return signal can be weak, sometimes less than a single photon per pixel. In addition, the pixel circuit is called upon to perform high-temporal-bandwidth time stamping. For linear-mode detectors and analog preamplifiers, sensitivity and speed are conflicting requirements. High noise bandwidth comes along with high signal bandwidth. The user of the system is forced to transmit enough photons so that the amplified return in each pixel can be discriminated from the noise floor. For a given amount of average laser power, this mandates low pulse repetition frequency with high energy per laser pulse.

The use of a Geiger-mode detector array offers a solution. The GMAPD is triggered by a single photon to produce a digital pulse, which is then digitally time stamped by the pixel circuit. There is no tradeoff between sensitivity and timing resolution. The lidar system is operated to take advantage of single-photon time stamping. The laser is operated at high pulse repetition frequency and low energy per laser pulse, so that each pixel gets average returns of a fraction of a photon. The image is built up by combining multiple frames. This mode of operation has two advantages. First, no photons are “wasted” overcoming a noise floor; timing information is obtained from each detected photon. Second, if a pixel sees returns from multiple depths, a histogram of timing values can be built up that reproduces the temporal profile that would be seen by a linear-mode detector operating with a much stronger signal.

**Figure 7:**
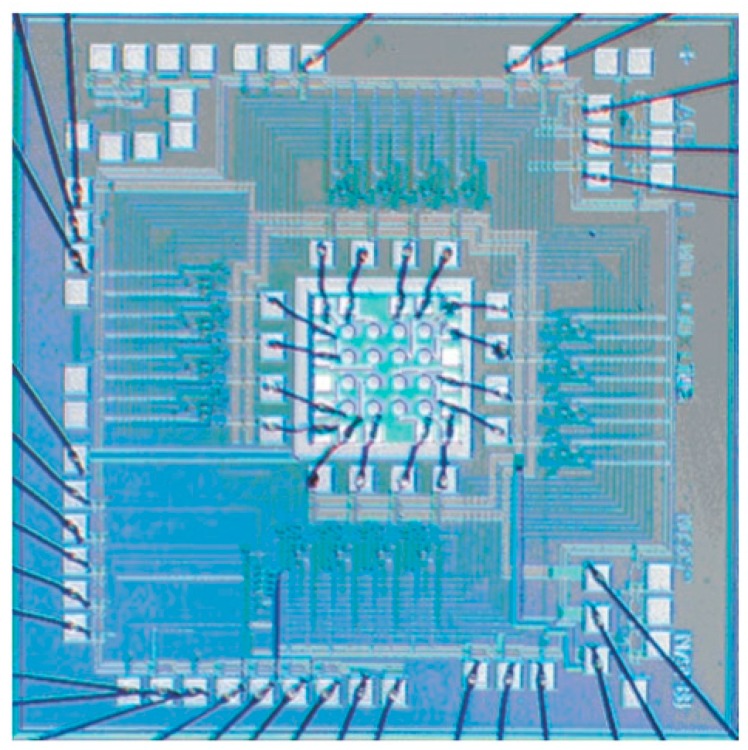
4 × 4 GMAPD array wire bonded to CMOS timing circuits.

The first proof of concept of a GMAPD array for lidar was a front-illuminated 4 × 4-pixel APD die piggy backed onto a 16-channel timing chip and the connections made with wire bonds. This device is shown in Figure 7. The CMOS circuit was fabricated through MOSIS using an HP 0.5-μm foundry process. The timing was done by broadcasting a clock to all the pixels, each of which had a pseudorandom counter based on a linear feedback shift register.

**Figure 8:**
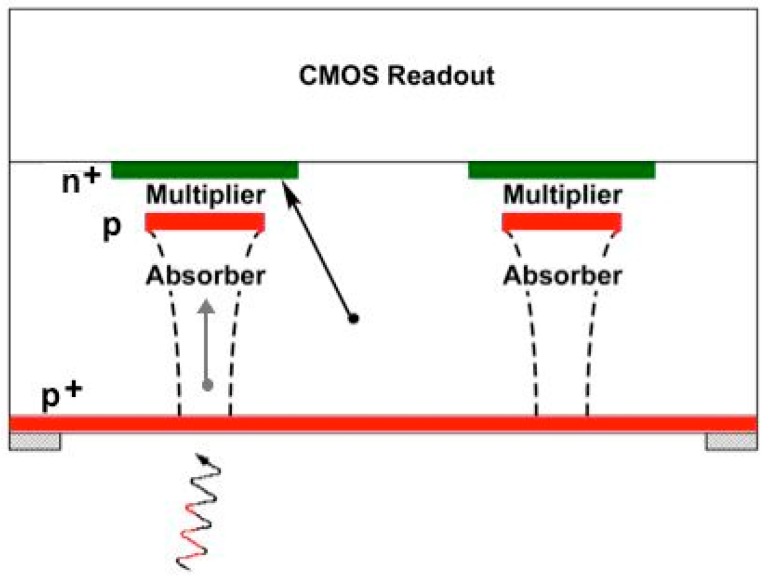
Low-fill-factor APD design used in lidar sensors.

The APD doping profile used in the lidar devices gives a low fill factor. A cross section through two adjacent pixels is shown in Figure 8. The center of the APD has the separate-absorber-multiplier structure depicted in Figure 2, but the *n*^+^-doped region extends out beyond the multiplier, creating a peripheral portion of the junction that collects electrons thermally generated in the region between pixels; these electrons do not trigger Geiger-mode events. A microlens array is integrated on the back side to concentrate incident light onto the responsive portions of the APDs. Alternatively, the lidar transmitter can be designed to project an array of spots onto the scene, which are then imaged on the APDs.

**Figure 9:**
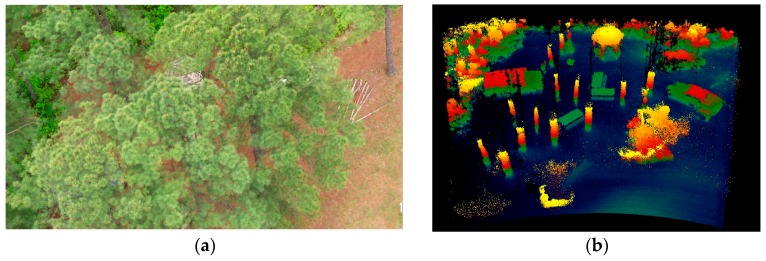
(**a**) Forest canopy as viewed from a nearby tower (**b**) Composite lidar image obtained by combining data collects from four different heights and then filtering out the early returns.

Following this proof of concept, the Laboratory developed a series of 32 × 32 arrays bridge bonded to MOSIS-fabricated CMOS circuits, and used them to build systems to perform foliage penetration and terrain mapping. Figure 9 shows the results of a foliage penetration experiment [15]. Lidar data was collected through a forest canopy from different heights on a tower. These multiple views enable returns to be collected from different angles through gaps in the foliage, thereby filling in the details about objects obscured by the foliage in conventional imagery. Figure 9b is a composite 3D image with the early returns from foliage filtered out, revealing vehicles, picnic tables, and a gazebo.

Lincoln developed the Airborne Ladar Imaging Research Testbed (ALIRT) system, an airborne lidar for terrain mapping. Initially, the system operated at 780 nm and used a 32 × 32 silicon GMAPD array. Because of the availability of efficient lasers at 1060 nm, however, the Laboratory developed short-wave-IR-sensitive GMAPDs based on InGaAsP detectors grown on InP substrates. These APDs also have the separate-absorber-multiplier structure, but the doping profile is created in the epitaxial growth and the pixels are isolated by mesa etch. 128 × 32 lidar image sensors were built by bump bonding the APD arrays to a CMOS timing circuit. Because of the exquisite sensitivity of the GMAPDs, the ALIRT system could collect wide-area terrain maps fifteen times faster than commercially available mapping lidars.

**Figure 10:**
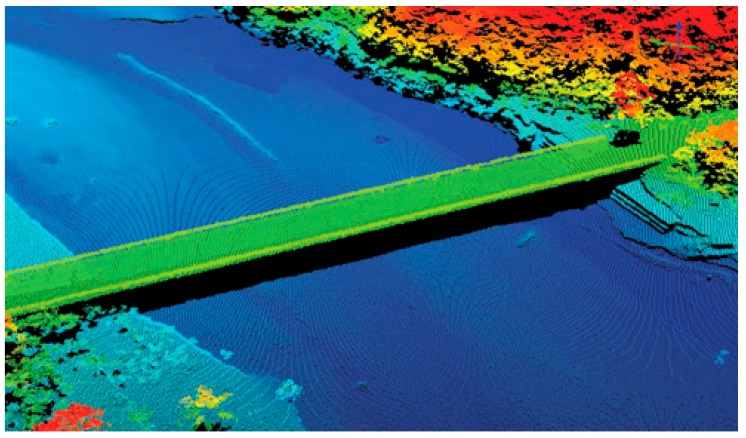
Lidar image of a bridge in Port-au-Prince, Haiti.

One example of the many missions flown by ALIRT was in support of humanitarian efforts in Haiti after the 2010 earthquake [16]. Figure 10 shows a lidar image of a bridge in Port-au-Prince, Haiti. ALIRT collected terrain maps of the city over time, which enabled relief workers to know where tents were being erected or taken down, what roads were blocked, and where it was safe to land helicopters.

A successor to the ALIRT system has an area coverage rate of 400 km^2^/h at 25-cm ground sampling distance. It can rapidly map a broad region and supply detailed three-dimensional images of every terrain feature or manmade structure over which it flies. It sees through foliage or dense dust clouds [17].

**Figure 11:**
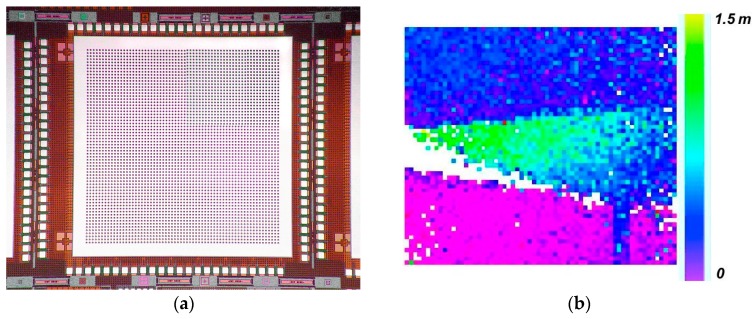
(**a**) 64 × 64-pixel lidar focal plane fabricated by 3D integration of a GMAPD array with two tiers of SOI CMOS circuitry (**b**) Lidar image of a cone obtained by illumination from a doubled Nd:YAG microchip laser.

Lincoln demonstrated another lidar focal plane that is highly significant from a technology evolution standpoint. Figure 11a shows the 3D-integrated 64 × 64-pixel lidar focal plane whose three-tier pixel cross section is shown in Figure 5. Figure 11b is a lidar image of a cone obtained using this device. To our knowledge, this was the first demonstration of a three-tier integrated circuit of any kind, based on a process that supports dense, arbitrarily placed micron-scale inter-tier connection vias (as opposed to chip stacking with peripheral wire bonds or ball grid arrays).

3D integration, as already pointed out, allows one to mix different technologies and put more circuitry within the area of a pixel. More importantly, however, it enables new imager architectures. Raw pixel data flows in parallel up through tiers of on-focal-plane processing circuits that extract information and reduce readout bandwidth.

## 5. A Photon-Counting Wavefront Sensor

**Figure 12:**
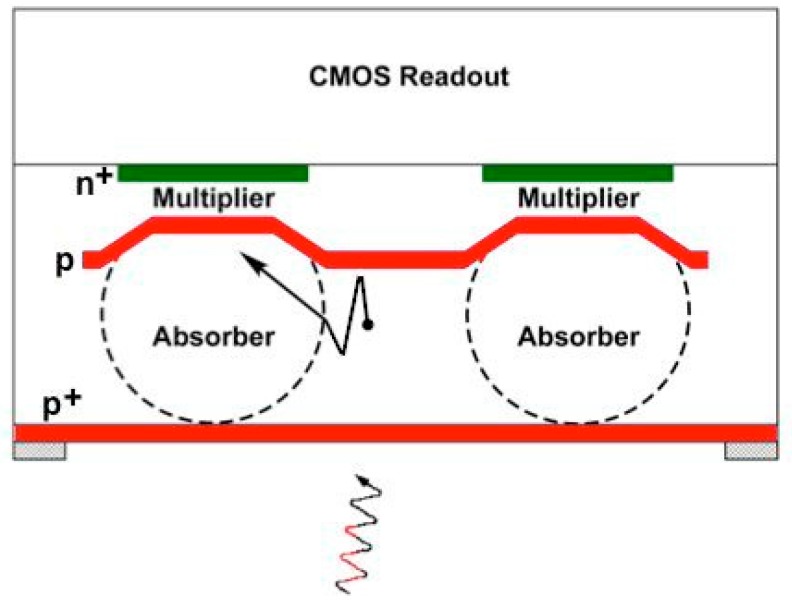
High-fill-factor APD design used in quad cells and passive imaging devices.

Adaptive optics systems for ground-based astronomy and space surveillance require sensors to measure the distortion of a wavefront (from either a bright star or an artificially created beacon) due to atmospheric turbulence. The Shack-Hartmann technique [18] uses arrays of lenslets that focus the light on quad-cell detectors; the displacement of a light spot from the center of a quad cell determines the partition of intensity among the four pixels. This in turn indicates the local wavefront tilt. Lincoln Laboratory has used its CCD imagers to build Shack-Hartmann wavefront sensors. However, in scenarios with weak beacon signals and fast wavefront update rates, the performance of CCDs is limited by readout noise. To address this problem, Lincoln developed Geiger-mode quad-cell arrays, to measure the number of photons from a beacon. Since the quad cell must be responsive to photons incident in between pixels, the low-fill-factor design shown in Figure 8 is not suitable. A high-fill-factor design was devised [19] and its cross section is shown in Figure 12.

The upper p-type layer is implanted at high energy through an oxide mesa so that the doping profile peaks at a relatively shallow depth (1 μm) in the center of each diode and deeper (2 μm) around the periphery and between pixels. The shallow portion of this stepped implant separates the absorber and multiplier portions of each detector. The step lowers the electric field at the periphery of the diode, preventing edge breakdown. The peripheral part of the diode functions as a guard ring, collecting surface-generated dark current without triggering Geiger-mode events. The deep portion of the implant, which is partially undepleted, prevents the guard ring from collecting photoelectrons generated in the absorber; as indicated in the figure, these photoelectrons reach a nearby multiplier region by a combination of diffusion and drift.

16 × 16 and 32 × 32-subaperture quad-cell arrays were fabricated and hybridized to readout chips that count the number of detection events with a 10-bit counter in each pixel. The device could be operated with 20-μs wavefront update latency while introducing no readout noise. Hybridization was accomplished first by bridge bonding and then later on by transfer and bump bonding. Pixel pitch within each quad cell was 50 μm, with a 200-μm spacing between subapertures [20].

**Figure 13:**
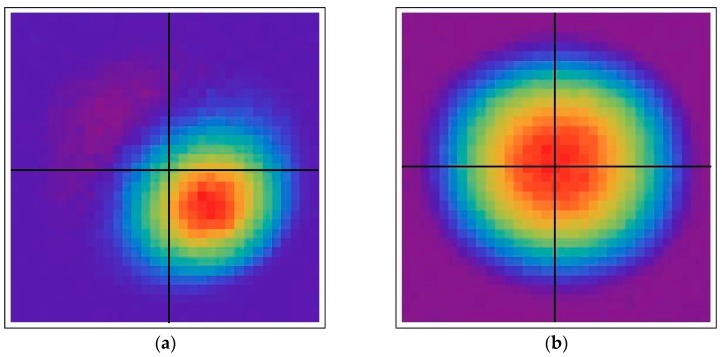
(**a**) Color contour plot of the count rate from the lower right detector as a function of the position of a small light spot raster scanned over the area of the quad cell; (**b**) Contour plot of the sum of the count rates from all four pixels as a function of light spot position.

To verify the functionality and contiguous spatial response, a focused 5-μm-diameter spot from a blue (450-nm) LED was raster scanned over the area of a quad cell and, at each 5-μm step, recording the photon counts from each of the four pixels. Figure 13a is a contour plot of the count rate of the lower right pixel as a function of the position of the light spot. Figure 13b shows the aggregate count rate from all four pixels. This data shows a monotonic transition of detection activity from one pixel to its neighbor as the light spot is being moved across the midpoint. It also shows no droop in the aggregate response in the central region.

These quad-cell arrays report out raw pixel intensity values, and the wavefront tilt calculation is done off chip. However, one can envision incorporating computational functions such as centroid and tracking into the pixel circuitry.

## 6. Passive Imaging

**Figure 14:**
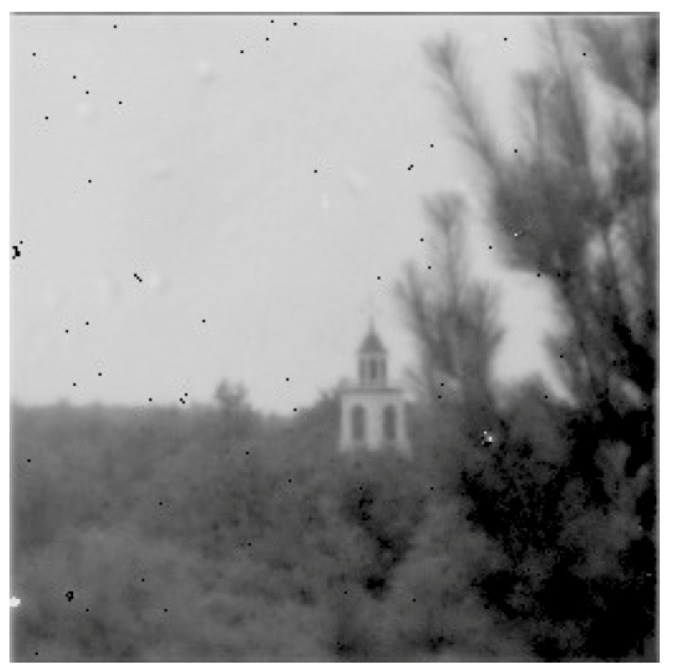
Image of church steeple.

In 2011, the Laboratory demonstrated a 256 × 256 passive photon counting imager with 25-μm pixel pitch [21]. The GMAPD array was based on the high-fill-factor design (see Figure 12) and was integrated to a foundry-fabricated CMOS readout using the transfer and bump bonding technique. To our knowledge, this was the first ever back-illuminated passive image sensor with this large a format based on hybridization of a GMAPD array to a CMOS readout. Figure 14 shows one of the first images taken with the device, a church steeple located about 3.5 km from the camera system. The black specks in the image are bump bond defects. Use of a 3D-integration technique results in far better image cosmetics.

The GMAPDs are armed, queried, and reset under supervision of external polling clocks. The CMOS pixel circuit has two readout modes. On the one hand, one can read out binary images in which each pixel reports whether or not it had a detection event since the last readout. This readout mode is called binary readout mode. On the other hand, one can read out the overflow bit of a 7-bit pseudorandom counter in each pixel; if set, this bit represents 127 cumulative detection events and it is reset by the readout operation. This readout mode is called overflow readout mode. At the conclusion of multiple overflow-bit readouts, the entire 7-bit counter can be read out to get the remainder. Binary readout mode and overflow readout mode are implemented by separate clocking systems that address rows or columns, respectively, in a rolling readout that does not blind the detectors. The overflow-bit readout mode provides for readout bandwidth reduction and dynamic range extension without having to put a large counter in the pixel. The overflow bits can be streamed to an Field-Programmable Gate Array (FPGA) that effectively provides the most significant bits of a longer counter. The image in Figure 14 was obtained by operating a 8 kiloframes/s in binary readout mode, digitally adding many frames off chip and performing a flat field correction.

**Figure 15:**
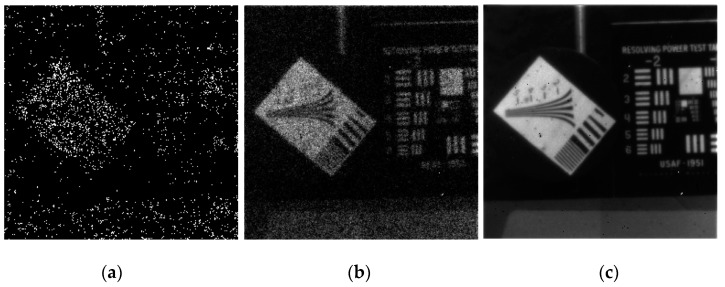
(**a**) One binary frame; (**b**) the digital sum of 64 binary frames; and (**c**) the digital sum of 32,768 binary frames.

Figure 15 shows images resulting from post-readout digital summation of short-time binary frames. Since there is no readout noise in a photon counting device, there is no noise penalty for such post-readout summation. One can imagine processing the binary frames before summing them to correct for scene motion or platform vibration. Even if each binary frame has a signal level less than one photoelectron per pixel, enough pixels have events to create discernable spatial structure, making such “smart integration” possible. This would not be feasible with a conventional analog imager, as even 1 electron rms readout noise would be too much.

A technological challenge with dense-pitch Geiger-mode APD arrays is optical crosstalk. During a detection event, the carriers traversing the avalanche region lose some energy by optical emission, and the near-infrared photons given off can spuriously trigger neighboring pixels. The quad-cell arrays [22] and the passive imager yielded valuable data on this phenomenon. Above a certain bias voltage, crosstalk-induced events dominate the dark count rate. Optical-crosstalk-based analytical models and Monte Carlo simulations match the spatial, temporal, and statistical characteristics of the dark count activity observed in the image data. The APDs in the passive imager did not have aggressive crosstalk reduction features, because that would have added yet another element of yield risk in the effort to prove out a new hybridization technique. The devices are operated at sufficiently low bias to avoid the crosstalk-dominated dark-count-rate regime. This limits the photon detection efficiency to the 10%–20% range. With the maturation of 3D integration techniques, current efforts are focused on crosstalk reduction.

## 7. The Future of GMAPD Imager Technology

The past 20 years have seen great progress in solid-state image sensors based on custom Geiger-mode avalanche photodiode arrays hybridized or 3D-integrated to all-digital CMOS readouts. Application to lidar represented the “low-hanging fruit,” as compelling system functionality could be realized with small imager format, coarse pixel pitch, low fill factor, and dark count rates in the tens of kcounts/s. Passive imaging is far more demanding of APD performance and, for back-illuminated silicon arrays, much more dependent on the maturation of hybridization techniques. Now that 3D integration methods have become available, optical crosstalk reduction is the next task. The successful demonstration of a 256 × 256 passive photon counting imager without crosstalk reduction is an encouraging result. A number of measures, such as capacitance scaling and low-reflectivity contact metal, can now be implemented to improve sensitivity.

All-digital CMOS tiers can exploit Moore’s Law scaling to realize increasingly sophisticated on-focal-plane processing functions. For lidar, these include in-pixel histogramming, tracking of objects of interest, multi-frame coincidence to reject background, and data thinning. For passive imaging, these include image stabilization, temporal change detection, and spatial filtering. Ultimately, one could implement a deeply scaled CMOS tier that could be programmed like an FPGA and support multiple firmware-defined functions with no hardware redesign.

While the focus of this review is imaging, GMAPD arrays sensitive in the short-wave infrared have potential as agile laser communications receivers. By spreading the received laser flux over multiple pixels, the effective APD reset time can be much shorter than the single-detector reset time, enabling high-data-rate free space optical communications links. In a remarkable technology demonstration, researchers at the Laboratory demonstrated the feasibility of an optical data link from a science satellite orbiting Mars. The laboratory demonstration used a pulse-position modulated format and error correction coding to achieve 0.5 photons/bit [23].

An imaging system traditionally consists of three distinct subsystems: (1) A bulky optical train that merely carries out an isomorphic transformation from object space to image space; (2) an image sensor that produces a high-aggregate-bandwidth stream of raw image data; and (3) a post-processing system to extract information of interest. Lincoln Laboratory’s long-term vision is to merge these functions, so that the work of information extraction is carried out by co-designed computational optics and smart focal planes. The use of coded apertures or light-field camera architectures can be combined with digital time stamping of photon arrivals to extract information about a scene, including regions that are obscured from the view of a conventional camera. The shift of computation burden to the optics and imager could also reduce readout bandwidth and enable low size, weight, and power.
